# Anti-Inflammatory and Pain-Relieving Effects of Arnica Extract Hydrogel Patch in Carrageenan-Induced Inflammation and Hot Plate Pain Models

**DOI:** 10.3390/pharmaceutics17020171

**Published:** 2025-01-28

**Authors:** Sang Gil Lee, Eun Byul Lee, Tack Soo Nam, Sunho You, Dahye Im, Kyusun Kim, Bonseung Gu, Ga-young Nam, Hyerim Lee, Soon Jae Kwon, Yun Seok Kim, Sang Geon Kim

**Affiliations:** 1 Center of Research and Development, A Pharma Inc., Goyang-si 10326, Gyeonggi-do, Republic of Korea; david_lsg@apharma.co.kr (S.G.L.); silverstar529@apharma.co.kr (E.B.L.); 2 R&D Center, Wooshin Labottach, Digital-ro 288, Guro-gu, Seoul 08390, Republic of Korea; wooshinnam@hanmail.net (T.S.N.); dbtjsgh34@nate.com (S.Y.); wooshinlabo-rdb@wooshinmed.com (D.I.); 0107167@naver.com (K.K.); bonseung321@naver.com (B.G.); nnngayoung@gmail.com (G.-y.N.); dgf506@naver.com (H.L.); 3 College of Pharmacy and Integrated Research Institute for Drug Development, Dongguk University-Seoul, Goyang-si 10326, Kyeonggi-do, Republic of Korea; ksoonj1112@gmail.com; 4 College of Pharmacy and Research Institute of Pharmaceutical Sciences, Seoul National University, Gwanakro-1, Gwanak-gu, Seoul 08826, Republic of Korea; bonobono670@snu.ac.kr

**Keywords:** arnica patch, edema, anti-inflammatory, inhibitory effects, pain relief

## Abstract

*Arnica montana* (AM), which belongs to the daisy family Asteraceae, has a longstanding traditional use in Europe and North America for pain and inflammation treatment. This study investigates the inhibitory effects of ‘*Arnica montana* extract hydrogel patch (AHP)’ on Carrageenan-induced paw edema and hot plate-induced pain models. AHP exhibited transdermal permeability without the occurrence of issues like crystal precipitation. This study employed two animal model assessments using AHP, in comparison with Arnicare Gel (AG), to evaluate anti-inflammatory and pain relief effects. AHP treatment for 2 days showed a decrease in paw edema thickness in mice as compared to vehicle or AG groups; Carrageenan-induced swelling increased maximally at 1 h with the AHP group demonstrating a higher reduction. Thus, the AHP group exhibited a lower ratio of right/left paw thickness and a superior reduction in swelling, supportive of its ability to diminish edema. A histological analysis showed that AHP treatment reduced inflammatory cell infiltration. Consistently, the mRNA levels of inflammatory markers (*tnfa*, *il1b*, and *il6*) were decreased to a greater extent than the AG group. Particularly, *tnfa* inhibition was better in the AHP group, and the levels of *il1b* and *il6* transcripts showed ~80% and 40% lower. Likewise, AHP reduced pain scores in a hot plate-induced rat model, although AG failed to do so. Together, these results demonstrate that AHP has long-lasting inhibitory effects on fluid effusion and edema formation, the production of inflammatory mediators, and pain-sensation, supporting its anti-inflammatory and pain-relieving pharmacological effects.

## 1. Introduction

Plant-derived medications are gaining prominence due to their cost-effectiveness and minimal side effects. *Arnica montana* (AM) is a flower belonging to the Asteraceae family and the Arnica genus. Traditionally, AM has a longstanding use in Europe and North America for treating pain and inflammation [1,2]. Various parts of AM including leaves, sepals, stems, flowers, roots, fruits, and branches have been employed with a focus on the whole plant. In European folk medicine, the flowers and rhizome have been used as a universal remedy since ancient times. Nowadays, it has been claimed that AM may serve as a hemostatic agent for conditions such as angina, vasodilation, the relief of vascular spasms, and the treatment of bruises and piles.

Inflammation and pain, as complex symptoms affected by physiological, social, and psychological factors [3], contribute to health issues and adversely impact patients’ quality of life [3,4]. Analgesics including narcotic analgesics, non-narcotic analgesics, and analgesic adjuvants, show clinical efficacy in relieving pain. Nevertheless, their utilization is limited due to adverse effects [5,6]. Pro-inflammatory cytokines, implicated in a variety of pathological conditions, play a role in inflammatory responses and pain sensitization [7]. Specifically, major cytokines such as TNFα, IL-1β, and IL-6 are closely linked to pathological processes and the consequential tissue damage [7]. Despite the above-mentioned analgesic effects of AM, there is limited information available on its effect on pain in the context of the regulation of inflammatory mediators.

With a variety of pharmacological properties such as analgesic, antibacterial, and anti-inflammatory activities, several formulations comprising AM are now available as forms of gel, cream, liquid, and tablets [8,9]. Unfortunately however, oral administration is limited due to its cytotoxic nature, and thus topical administration, particularly in the form of gel or cream, is common [2]. Nonetheless, topical administration using gel or cream formulations faces limitations due to clothing and activities, potentially impacting drug penetration. When the component is applied as a dry patch, the adhesiveness is low because the surface is oily. Moreover, AM extract has the disadvantage of being weak against moisture.

Plasters are categorized into moist poultices called cataplasms and solid formulations known as plasters, both in the form of a dressing. Cataplasms, due to their nature, include a flexible layer separate from the adhesive layer [10]. Plasters, containing acrylic resins, for example, do not have moisture, presenting a disadvantage in terms of skin irritation compared to cataplasms [11]. To overcome the drawbacks of these traditional cataplasms and plasters, as well as cream formulations, there is ongoing research into hydrogel dressings as a transdermal absorption formulation. Herein, we report the development of ‘AM-containing hydrogel patch’ and its pharmacological effects, based on our preliminary research outcomes, which suggests that this formulation proposes a solution to the above problems and improves adhesion for the stable penetration of the active components.

This research also explored the efficacy of the AM hydrogel patch formulation on the edema formation, the production of inflammatory mediators, and pain sensation. Specifically, we sought to evaluate the effects of the AM hydrogel patch on the levels of inflammatory markers using the Carrageenan-induced inflammation model, examining its inhibitory effects on (1) edema formation, (2) inflammatory cell infiltration, and (3) inflammatory markers. Further, we utilized a rat model to assess its pain-relieving effect in comparison with a conventional gel formulation. The outcomes of this study show the evidence that the AM hydrogel patch has bona fide anti-inflammatory and pain-relieving effects, and more intriguingly, the hydrogel formulation exerted a convenient long-lasting biological effect compared to a gel-type formulation.

## 2. Materials and Methods

### 2.1. Preparation and Manufacture of the AM Extract Hydrogel Patch

The hydrogel patch pharmaceutical composition containing AM extract was developed, with the material composition summarized in Table 1 (Wooshinlabottach Co., Ltd., Seoul, Republic of Korea). The AM Patch was designed to allow the uniform distribution of high concentrations of active ingredients. The pH of the AM extract hydrogel patch ranged from 5.0 to 6.0, exhibiting transdermal permeability without issues such as crystal precipitation during manufacturing and storage (Table 1).

### 2.2. Mouse Experiments

The animal care and studies were reviewed and approved by the Institutional Animal Care and Use Committee (IACUC) at Dongguk University (No. IACUC-2021-035-2, 10 June 2021). The C57BL/6 mice were purchased from Jabio (Suwon, Republic of Korea), housed at standard temperature (22 ± 2 °C) and humidity (50 ± 5%) under a 12 h/12 h light/dark cycle, pathogen-free air, with food and water available ad libitum. Male mice at 8 weeks of age were used. To minimize environmental differences, mice were maintained for at least a week before each experiment.

### 2.3. Carrageenan-Induced Edema Assays

Edema was induced using 1% Carrageenan. A total of 50 μL of 1% Carrageenan in saline solution was injected into the right paw of the mouse and 50 μL of saline into the left paw. The left paw was injected with 50 μL of saline for mock treatment (for comparative purposes, the opposite paw was selected as the observation target, inducing swelling in the mouse’s paw). The AM hydrogel patch (Wooshin Labottach Co., Ltd., Seoul, Republic of Korea) or Arnicare gel (Bioron Co., Ltd., Newtown Square, PA, USA) was applied 2–3 times daily, depending on the characteristics of the test substance. The formulations were administered at 9:00 a.m. every day for 2 days, and they were applied immediately to enhance application stability. This step was repeated with an additional application at 1:00 p.m. (plus 6 p.m. for gels) (i.e., 2 or 3 times daily). The AM Patch was replaced at 9 p.m. On day 3, the sizes of the edema were measured and photographed at 1, 2, and 3 h time points after Carrageenan administration and the final AM hydrogel patch or Arnicare gel application; the morphological changes were observed hourly, which was terminated when changes were recognized.

### 2.4. Preparation of Paw Tissue Samples

The mice were euthanized and sacrificed through cervical dislocation to obtain paw samples. The left paw tissues were removed from blood and fur from the skin. Kimwipes were used to eliminate moisture. Then, a razor blade was used to collect integumentary tissue from the swollen area of the mouse’s paw and the opposite side, which was subjected to immunohistochemistry and qRT-PCR assays for key inflammatory mediators.

### 2.5. Histopathology Analysis

Mouse paw tissues were fixed in 10% formalin, embedded in paraffin, cut into sections, and mounted on slides. Paraffin-embedded colon tissue sections were stained with hematoxylin and eosin (H&E) using a commercial staining kit (ScyTek Laboratories, Logan, UT, USA) for tissue morphology.

### 2.6. RNA Isolation and Quantitative RT-PCR Assays

Total RNA was extracted using Trizol (Invitrogen, Carlsbad, CA, USA) and was reverse-transcribed. The resulting cDNA was amplified by qRT-PCR using LightCycler DNA Master SYBR Green-I Kit (Roche, Mannheim, Germany) according to the manufacturer’s instructions. Gapdh was used as a normalization control. The primer sequences used for qRT-PCR assays are listed in Supplementary Table S1.

### 2.7. Hot Plate Pain Scoring Test in Rats

Seven-week-old male SD rats were purchased from Orientbio (Seongnam, Republic of Korea) and housed at standard temperature (22 ± 2 °C) and humidity (50 ± 5%) under a 12 h/12 h light/dark cycle in Wooshin Labotach Co. The treatment doses of the AM hydrogel patch and the Arnicare gel were calculated based on the body surface area of rats compared to humans. A dose of 0.5 g, equivalent to 35 mg of active ingredients, was established for the Arnicare gel, whereas that of the AM Patch was conducted to have a dose of 3.46 cm^2^, equivalent to 3.46 mg. The rats were randomly assigned to four groups: AM Patch, 3.46 mg for 1 h (patch, 1 h; n = 8), AM Patch, 3.46 mg for 2 h (patch, 2 h; n = 8), Arnicare gel, 35 mg for 1 h (gel, 1 h; n = 8), and Arnicare gel, 35 mg for 2 h (gel, 2 h; n = 8). The formulations were applied on the left thigh of the animals. The formulations were removed 1 or 2 h afterward, and a hot plate test was conducted to evaluate the pain response. All animal experiments were approved by Wooshin Labottach Co., Ltd., Ethical Committee for Animal Experimentation (approval number; WS23002, 20 October 2023, WS23003, 18 November 2023).

Analgesic activity was assessed by the hot plate test, with the plate surface set at 55 °C, and the rat’s movement confined within an acrylic cylinder. Rats subjected to the administration of test substances were individually placed on the plate and observed for abnormal behaviors such as licking the hind paw, stamping, and jumping over a duration of 1 min. A pain score was assigned: 1, licking and stamping; and 2, jumping. If no response occurred within 30 s, the rat was removed from the hot plate to prevent a heat-related injury. Between each test, the hot plate surface was cleaned with 70% ethanol. The control value represents the mean pain score of vehicle-treated animals within each group. The pain score ratio compares the pain scores between the control group and the treatment group.

The hot plate test is a standard method for evaluating pain responses, providing a robust quantitative comparison of the analgesic effects. This method was chosen for its ability to measure pain-relief effects over time. Moreover, the hot plate test is a rapid and effective method for measuring acute thermal pain. Its sensitivity to analgesic effects makes it a choice for evaluating thermal pain and the efficacy of the hydrogel patch. However, it must take into account more complex behavioral characteristics compared to other pain assays. The latency time can be influenced not only by the analgesic effect but also by the rodent’s genotype or learning through repeated measurements [12,13]. This experiment was performed only once per subject to minimize the impact of behavioral changes due to learning. This study did not consider the maximum and minimum values for data reliability.

### 2.8. Statistical Analysis

The significance of differences among groups was analyzed by one-way analysis of variance (ANOVA) with Tukey’s multiple comparison test using GraphPad Prism version 7.03 (GraphPad Software Inc., San Diego, CA, USA). The data were shown as the mean ± standard error of the mean (SEM). Statistical significance was set at *p* < 0.05.

## 3. Results

### 3.1. AM Extract Hydrogel Patch Formulation and Patch Preparation Process

**Figure 1:**
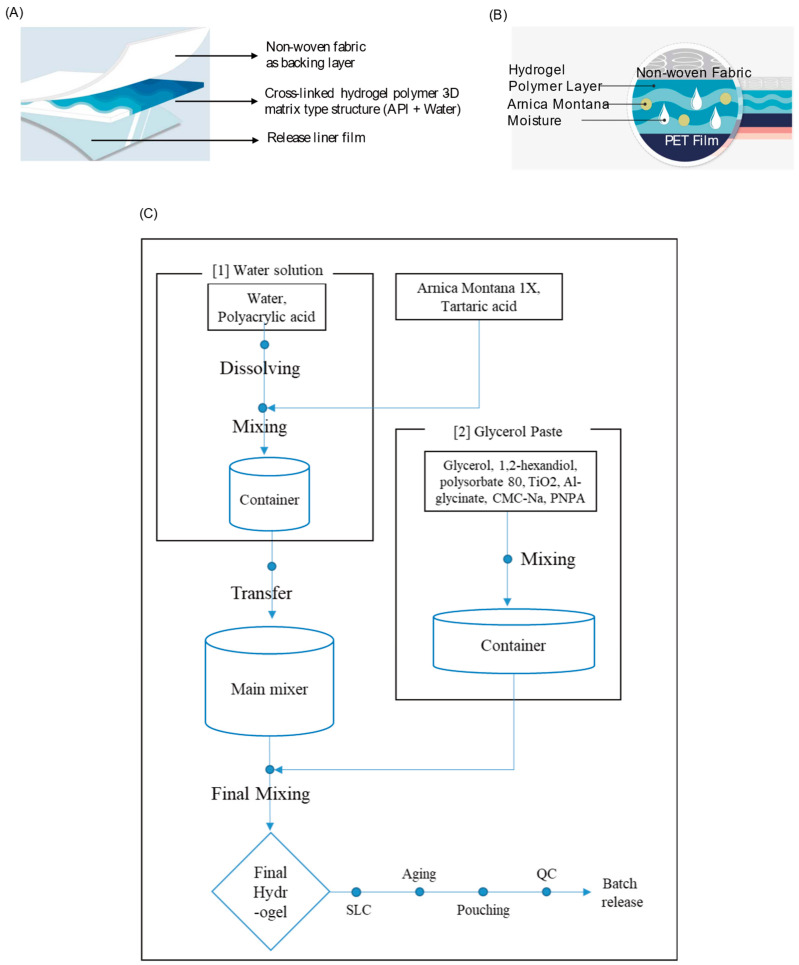
Schemes depicting the layers of the AM extract hydrogel patch and the formulation process chart. (**A**) A schematic illustrating the layers of the AM extract hydrogel patch. (**B**) An active component layer depicting hydrated hydrogel polymer and an active ingredient. (**C**) A patch preparation process flow.

The formulation in this study utilized a hydrogel cataplasm, where water is the main component (Figure 1A,B). This ensures excellent adhesiveness and allows the penetration of a significant amount of active ingredients for an extended period. As part of the patch preparation process, one portion of *Arnica montana* mother tincture was diluted with nine portions of water to prepare *Arnica montana* 1X. For the water solution, polyacrylic acid was dissolved in water, followed by the addition of *Arnica montana* 1X and tartaric acid, which were mixed thoroughly. The glycerol paste was prepared by mixing glycerol, 1,2-hexanediol, polysorbate 80, titanium dioxide, aluminum glycinate, carmellose sodium, and sodium polyacrylate. This paste was then combined with the water solution to form a hydrogel. The hydrogel was spread between nonwoven fabric and a PET film, laminated, and cut into patches measuring 10 cm by 12 cm. These patches were aged for 24 h and stored in aluminum foil pouches. Finally, the product was analyzed to ensure it met specifications, including the identification of chlorogenic acid (Figure 1C).

### 3.2. Inhibition of Carrageenan-Induced Edema Formation

**Figure 2:**
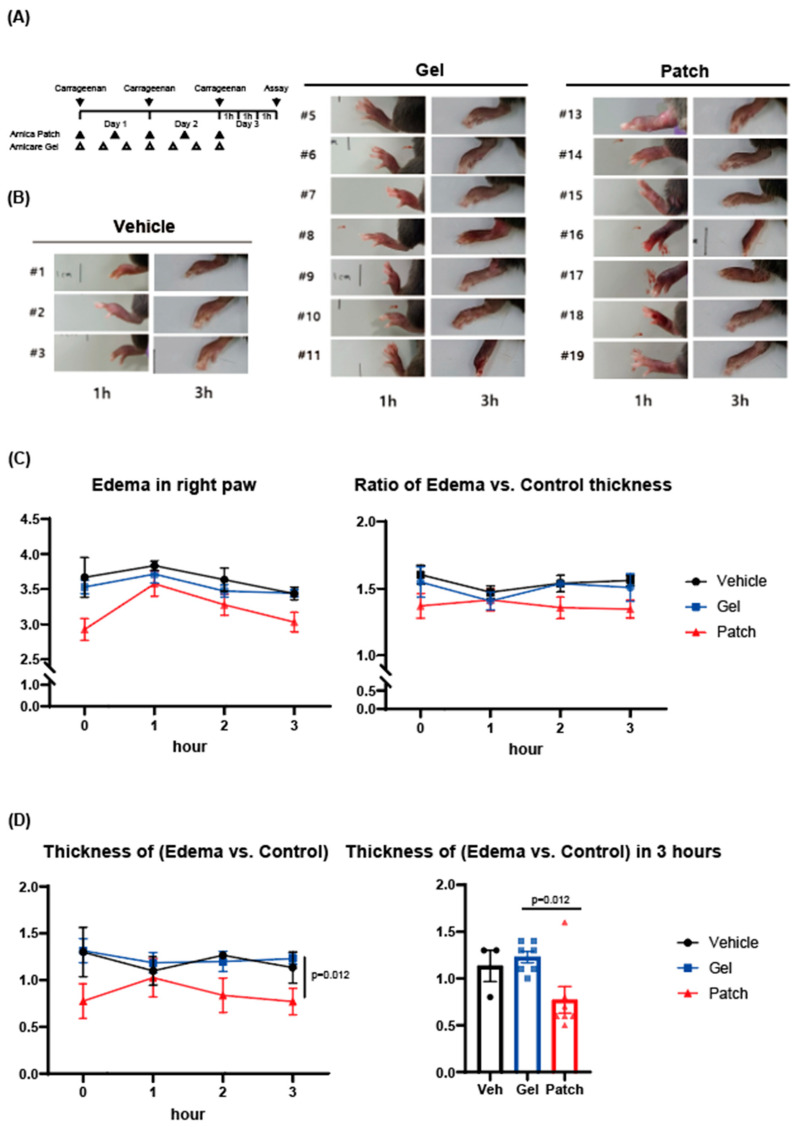
Inhibitory effects of AM extract hydrogel patch on Carrageenan-induced edema formation in mice. (**A**) Experimental scheme. (**B**) Appearances of paw edema in each group. (**C**) Left: Thickness of the right paw induced with Carrageenan for edema formation. Right: Ratio of the thickness between the right paw induced with Carrageenan and the left paw treated with saline (Control). (**D**) Left: Time-dependent difference in thickness between the right paw induced with Carrageenan and the left paw treated with saline (Control). Right: Difference in thickness at the 3 h time point between the right paw induced with Carrageenan and the left paw treated with saline (Control). Group 1 (n = 3), Carrageenan + saline (Vehicle); Group 2 (n = 7), Carrageenan + Arnicare Gel (Gel); and Group 3 (n = 7), Carrageenan + Arnica Patch (Patch).

As an effort to assess the pharmacological effect of the newly developed AM hydrogel patch on inflammation, we employed the Carrageenan-induced paw edema model. To assess its biological effect, we injected Carrageenan injection into mouse paws, and we first examined morphology. The size of the fluid effusion and swelling elicited by Carrageenan increased at the 1 h time point and then gradually decreased afterward (Figure 2A). When comparing the thickness of the right paw induced by Carrageenan, the AM Patch group treated for the first 2 days showed a decrease in the thickness of the initial paw edema induced by Carrageenan administration at the third day, compared to the vehicle group. Moreover, it was confirmed that the AM Patch group had a more significant reducing effect than the Arnicare gel treatment group (Figure 2B).

When compared not only based on the thickness difference in the right paw induced by simple edema but also by comparing the ratio with the left paw treated with saline, the AM Patch group exhibited a lower ratio in the size of the swelling than either vehicle or Arnicare gel treatment groups. Please note that the reason for the decrease in the right/left ratio at the 1 h time point was attributed to the temporary increase in thickness due to saline administration in the left paw, causing an increase not related to swelling but to the volume of saline (Figure 2C).

A similar method of re-analysis based on the difference in thickness between the left and right paws showed consistent results. In the final assay stage (i.e., the 3 h time point on the third day), the left/right paw thickness difference was similar for vehicle and Arnicare gel treatment groups, whereas the AM Patch treatment group showed a statistically significant difference, compared to the Arnicare gel treatment group (*p* = 0.012). Fluid effusion due to edema formation was confirmed by measuring the thickness of the paw. As previously mentioned, the size of edema induced over the long term was the lowest in the AM Patch group. Furthermore, regarding the changes in short-term paw edema on the third day, the AM Patch group showed a higher effectiveness compared to the Arnicare gel group (Figure 2D).

The experimental design utilized a Carrageenan-induced paw edema model, with Carrageenan injected into the right paw and saline into the left paw. Treatment groups included the hydrogel patch, gel, and vehicle, applied consistently over 2 days. Paw thickness was measured at multiple time points (1, 3, and 24 h) post-Carrageenan injection. The hydrogel patch group exhibited the most significant reduction in paw thickness compared to the vehicle and gel groups. Statistical analysis using ANOVA confirmed that these differences were significant (*p* < 0.05), supporting the patch’s superior anti-inflammatory effect.

For C and D, values were expressed as mean ± SEM (* *p* < 0.05, ** *p* < 0.01). Statistical significance was tested via two-tailed Student’s *t*-tests.

### 3.3. Hematoxylin and Eosin Analyses of Paw Edema Tissue

**Figure 3:**
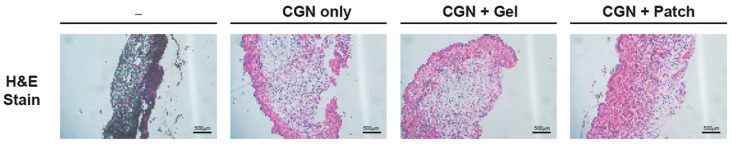
Histopathological Analysis of Paw Edema in the Carrageenan-Induced Inflammation Model. Representative H&E staining images of paw edema tissue in the Carrageenan-induced inflammation animal model.

In the histological examination (H&E staining) of the paw sections, Carrageenan injection resulted in edema formation, as evidenced by a wide and mild tissue composition overall in the microscopic visual fields. Consistently, a significant increase in neutrophils infiltration was evident in the Carrageenan treatment group. Notably, the Arnicare gel treatment group displayed suppression in this effect, demonstrating a clear inhibition of inflammatory cell infiltration from blood vessels. In this group, the tissue composition became denser with the efficacy of reducing inflammatory cell infiltration being more pronounced, compared to the other groups (Figure 3).

A histological analysis of paw tissue sections was conducted to assess inflammatory cell infiltration. Samples were prepared using standard H&E staining procedures. The vehicle group exhibited severe neutrophil infiltration and disrupted tissue organization, characteristic of acute inflammation. In contrast, the hydrogel patch group showed reduced cellular infiltration and improved tissue structure, suggesting an effective anti-inflammatory response.

### 3.4. Inhibition of Inflammatory Transcript Marker Levels

**Figure 4:**
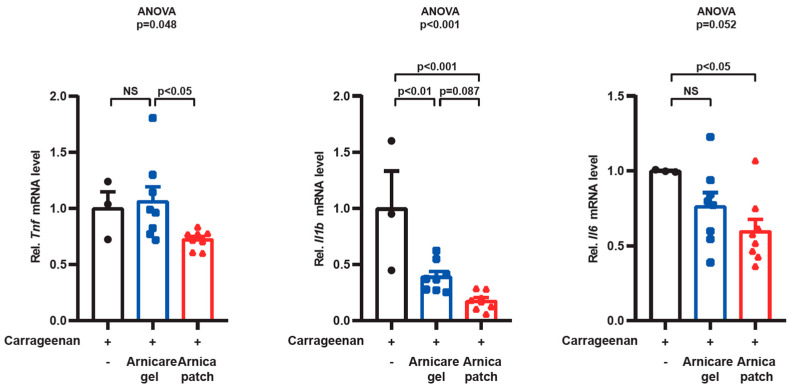
Inhibitory efficacy of AM extract hydrogel patch on inflammatory markers within paw edema in Carrageenan-induced inflammation mouse model. RT-PCR assays were conducted for *tnfa*, *il1b*, and *il6* mRNA levels in the tissue of the right paw induced with Carrageenan. Group 1 (n = 3), Carrageenan + saline (-); Group 2 (n = 7), Carrageenan + Arnicare Gel (Gel); and Group 3 (n = 8), Carrageenan + AM extract hydrogel patch (Patch). Statistical significance was tested via one-way ANOVA coupled with Bonferroni’s method or the LSD multiple comparison procedure when appropriate. Values were expressed as mean ± SEM. In the data representation, black dots denote Group 1, blue dots denote Group 2, and red dots denote Group 3.

Subsequently, RT-PCR assays were conducted on the inflammatory markers for *tnfa*, *il1b*, and *il6* using the right paw of the mice. To ensure the stabilized effects of the hydrogel patch and gel formulations, the day 3 time point was selected for inflammatory markers. Notably, the AM Patch group showed statistically significant ~40% lower *tnfa* levels compared to the Gel group. However, there was no significant difference in *tnfa* levels between the vehicle group (i.e., Carrageenan only) and the Arnicare gel treatment group. The expression levels of *il1b* were significantly lower by more than 60% in both the Arnicare Gel and Arnica Patch groups compared to the Carrageenan-only group (Figure 4). Again, the AM Patch treatment group demonstrated superior effectiveness, showing an approximately 80% inhibition. By the same token, the AM Patch treatment group exhibited statistically significant 40% lower expression in *il6* compared to vehicle-treated control. However, no significant difference existed between the vehicle and the Arnicare gel group. The paw of the saline vehicle group, where Carrageenan was not administered, did not induce swelling, and thus the inflammatory markers were almost non-existent.

### 3.5. Pain-Relief Effect of AM Patch in Hot Plate Test

**Figure 5:**
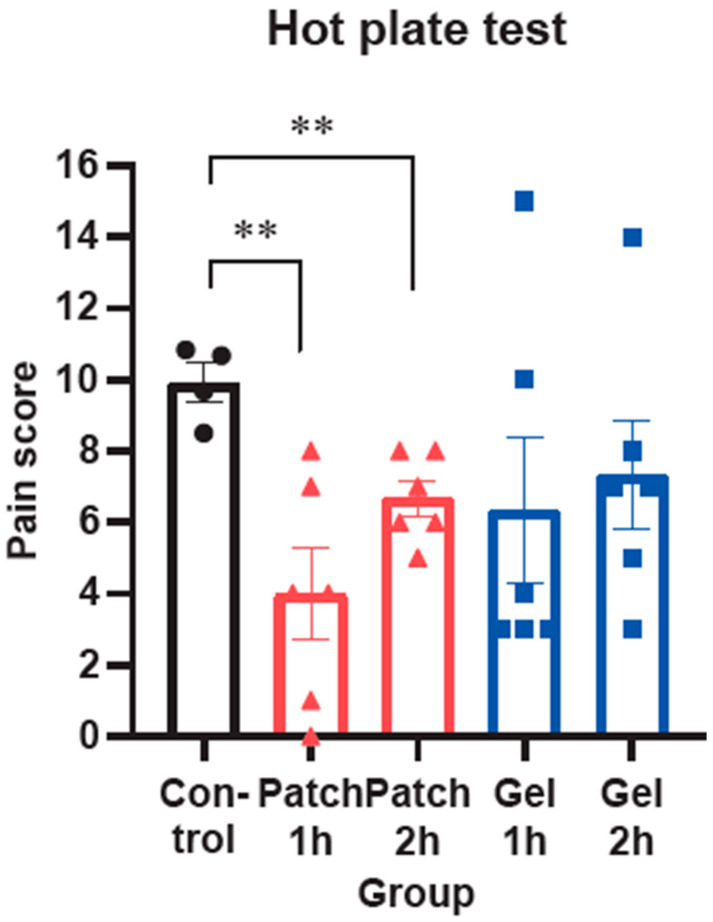
Pain-relief effect of AM extract hydrogel patch in hot plate test using rats. Rats were subjected to the hot-plate test, and pain scores were assessed 1 h or 2 h after AM extract hydrogel patch (Patch) or Arnicare gel (Gel) administration. The pain score ratio was obtained from those from the control group and the treatment groups (n = 6 rats per group). Values were expressed as mean ± SEM (** *p* < 0.01 vs. control). In the data representation, black dots denote the control group, red dots denote the Patch group, and blue dots denote the Gel group.

Having identified the bona fide anti-inflammatory effects of the AM Patch, we lastly examined its pain-relieving effect, as monitored by the hot plate test in rats. One or two hours after the AM Patch or the Arnicare gel administration, the hot plate test was conducted to assess pain scores in the control and the administration groups. As expected, the AM hydrogel patch treatments significantly reduced pain scores at either 1 or 2 h after treatment. However, the Arnicare gel treatment group failed to show significant changes as compared to the control (Figure 5).

In the cross-sectional H&E staining of the paw induced by Carrageenan (CGN), overall tissue organization was characterized by a significant increase in the infiltration of inflammatory cells (neutrophils) in the Carrageenan-only group. This phenomenon was suppressed in the AM extract hydrogel patch group (Patch), indicating an inhibition of inflammatory cell infiltration. According to the tissue image analysis of the AM extract hydrogel patch group, the tissue structure becomes denser, demonstrating a pronounced efficacy in reducing inflammatory cell infiltration compared to other groups.

## 4. Discussion

This study systematically explored the anti-inflammatory and analgesic potential of a hydrogel patch containing *Arnica montana* (AM) extract, emphasizing its superiority over a gel formulation through various pharmacological, histological, and molecular evaluations. The findings clearly demonstrate that the hydrogel patch offers distinct advantages in reducing inflammation, alleviating pain, and providing sustained therapeutic effects.

*Arnica montana* grows in alpine meadows, reaching a height of 20–30 cm. The leaves radiate from the root and are opposite on the stem. Branching occurs at the end of the stem, bearing 1–3 flowers. Blooming in June and July, the yellow flowers have a diameter of 6–8 cm, with a dense covering of bristles and linear patterns [9,14]. Both the flowers and the rhizome have a bitter taste and contain essential oils and resins. In traditional medicine, *Arnica montana* is utilized as a hemostatic agent for conditions such as angina, vasodilation, the relief of vascular spasms, and the treatment of bruises and piles [1,8]. Thus, the fermented extract of Arnica, with its anti-inflammatory and analgesic properties, can provide relief from pain in conditions such as bruises, contusions, muscle pain, joint pain, bone fracture pain, lower back pain, shoulder stiffness, neuralgia, rheumatic pain, skin itching, insect bites, and wound healing [1,8,15,16].

Recent studies, such as the one by [17], have highlighted the therapeutic potential of *Arnica montana* in reducing inflammation and managing pain through its active phytochemical components. This aligns with our findings that demonstrate the efficacy of a hydrogel patch formulation containing AM in reducing inflammatory markers such as TNFα, IL-1β, and IL-6. The anti-inflammatory properties have been attributed to active components like sesquiterpene lactones, which modulate the production of pro-inflammatory cytokines and inhibit NF-κB pathways. These mechanisms are consistent with the observed reductions in inflammatory markers, suggesting that the hydrogel patch actively delivers the bioactive components to the target site.

The conventional gel and ointment formulations are available for the local application of the active ingredients including sesquiterpene lactones, flavonoids, and volatile oils [16]. The AM hydrogel formulations comprised the following ingredients: polyacrylic acid partially neutralized as a hydrogel-forming agent; a crosslinking agent selected from the group consisting of aluminum salt, magnesium salt, and calcium salt; and a combination of an acid, with the composition further including 30–50% by weight of water. Herein, we used various organs or parts (e.g., leaves, sepals, stems, flowers, roots, fruits, and branches) of *Arnica montana*, with a focus on using the entire plant as the primary source.

The AM hydrogel patch composition was designed to enhance its physical and pharmacological properties; Glycerin serves as a humectant to improve hydration and penetration, while sodium polyacrylate contributes to viscosity regulation and patch adhesion. Tartaric acid maintains formulation stability, and 1,2-hexanediol acts as an antimicrobial agent to ensure product quality during storage. Together, these components would be of help to optimize skin adherence and sustained effect. Notably, the hydrogel patch composition has the ability to not interfere with daily activities after external application. Additionally, the hydrogel patch appears to overcome the drawbacks of conventional cataplasms and plasters, such as skin irritation, toxicity from residual solvents and/or unreacted monomers, and other problems associated with prolonged crosslinking (unpublished data, not shown).

Among the supplements, the main function of hydrogel patches is mostly a cooling and sedation effect called cryotherapy (common use of low temperature during treatment) [18]. The primary effect of cold in pain management is to lower the temperature of the damaged tissue, which reduces the metabolic rate of the tissue and helps the tissue survive after the damage. And the cooling effect can prevent internal bleeding due to the contraction of peripheral blood vessels. The AM hydrogel cataplasm patch formulation in this study provides a unique double action (cool and hot sensation); there is no actual temperature change in the skin, but instead, the neurotransmitter of the nerve acts, resulting in a perceived exothermic effect. Like the exothermic effect by vanillyl butyl ether (VBE), which is a stable ether, it directly stimulates receptors at the nerve end to create an exothermic feeling. In pain management, the primary effect of cryotherapy is to lower the temperature of damaged tissues, reducing the metabolic rate of the tissue and aiding in its survival after injury. Additionally, the cooling effect may prevent internal bleeding caused by the contraction of peripheral blood vessels. Thus, the main function of the AM hydrogel patch would be predominantly cryotherapy, which involves the therapeutic use of low temperatures. Consequently, the invented AM composition in the form of a hydrogel patch may have an advantage over gel formulation; the outcomes of the present study provide evidence that invented AM composition in the form of a hydrogel patch may be superior to gel formulation in the context of application and sustainability.

Carrageenan-induced paw edema was pharmacologically compared and observed from the perspectives of both inflammatory mediator production and fluid effusion. Both aspects increased due to Carrageenan induction, and it was also confirmed that both AM Patch and Arnicare gel groups exhibited inhibitory effects on edema. The AM Hydrogel Patch group displayed a notable reduction in paw edema thickness in mice when compared to the vehicle control or Arnicare gel groups, showing a lower thickness ratio and a more substantial reduction in swelling. Furthermore, histological analysis confirmed that AM Patch treatment reduced inflammatory cell infiltration which was superior to the Arnicare gel group.

Other assessments were also carried out using the formulations to comparatively assess their anti-inflammatory properties. In line with the above observations, the AM hydrogel patch applications significantly lowered mRNA levels of inflammatory markers (*tnfa*, *il1b*, and *il6*) compared to the AG formulation; In particular, the inhibition of *tnfa*, a mediator responsible for the most significant inflammation and the consequential cell death, was notably superior in the AHP group, with *il1b* and *il6* transcripts also showing lower levels. The increase in inflammatory mediators such as *il1b* showed a decreasing effect in both the patch-treated and gel-treated groups. Of note, *il6* showed an inhibitory effect only in the Patch group. The outcomes of this study offer a bona fide anti-inflammatory patch composition comprising Arnica’s extract as an active ingredient. This contention was fortified by the finding that AM hydrogel patch treatment diminished pain scores in a hot plate-induced pain scoring model. In this assay, Arnicare gel administration failed to do so presumably because of the lack of sustaining effect.

**Figure 6:**
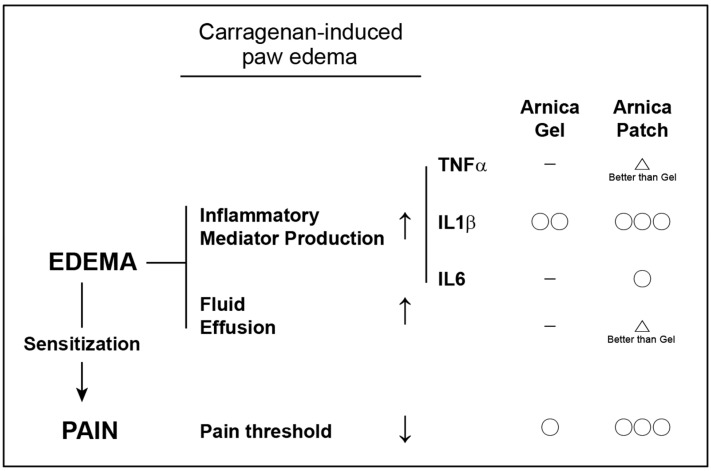
A diagram illustrating the anti-inflammatory and pain-relieving effects of AM extract hydrogel patch (Arnica Patch) and Arnicare Gel. Circles (○) indicate that the treatment demonstrates efficacy for the respective parameter, while horizontal lines (-) denote no observed effect.

The history of homeopathy traces back to the 18th-century German physicist Samuel Hahnemann, who postulated the principle “like cures like.” In the 19th century, Hugo Paul Friedrich Schultz hypothesized that the effects of consuming small amounts of toxins were opposite to those of consuming large amounts [19]. In 1888, he demonstrated that a slight concentration of yeast toxin could increase yeast growth a hundredfold [19]. As a result, these diverse observations were formalized by Ante in 1988, establishing the principles of early pharmacology in homeopathy. A pure plant-based homeopathic therapy containing Arnica extract has been locally used for pain management. Consistently, the results of this study confirm the anti-inflammatory and analgesic effects of the AM hydrogel patch. Specifically, the patch reduced the thickness of Carrageenan-induced paw edema, inhibiting the expression of inflammatory markers including TNFα, IL-1β, and IL-6, as also strengthened by the histological evidence of reduced inflammatory cell infiltration (Figure 6). In addition, the hydrogel patch exhibited pain relief in the hot plate test. These results support therapeutic advantages of the hydrogel patch.

## 5. Conclusions

Overall, our findings highlight the significant therapeutic potential of the *Arnica montana* hydrogel patch as a modern and effective approach to managing inflammation and pain. Compared to the conventional gel formulation, the hydrogel patch demonstrated superior efficacy in reducing Carrageenan-induced paw edema, suppressing inflammatory mediators, and alleviating pain in preclinical models. The robust inhibition of key cytokines such as *tnfa, il1b*, and *il6*, along with reduced inflammatory cell infiltration, underscores the patch’s potent anti-inflammatory properties.

While traditional *Arnica montana* formulations (e.g., gels, creams) face limitations such as rapid absorption and limited duration of action, the hydrogel patch offers a mechanism that ensures prolonged therapeutic effects. This addresses the challenges noted in prior studies, including short-lived efficacy and inconsistent drug delivery. The hydrogel patch’s innovative dual-action sensory effect, delivering both cooling and perceived exothermic sensations, enhances its therapeutic utility by improving user experience and augmenting pain relief. Its controlled release mechanism ensures the sustained delivery of active ingredients, making it particularly suitable for conditions requiring prolonged therapeutic action. Moreover, the patch formulation successfully addresses the limitations of traditional topical treatments, such as skin irritation, toxicity from residual solvents, and poor adhesion, offering a safer and more convenient option for patients.

Our findings also suggest that the hydrogel patch has potential for further optimization by incorporating additional active pharmaceutical ingredients, such as ketoprofen, ibuprofen, diclofenac, or lidocaine, to broaden its applications in pain and inflammation management. This adaptability positions the hydrogel patch as a versatile platform capable of addressing diverse clinical needs, from acute injuries to chronic inflammatory conditions.

In summary, our findings demonstrate that the *Arnica montana* hydrogel patch represents an advanced therapeutic modality that combines the traditional benefits of herbal medicine with modern pharmaceutical technology. Its ability to provide sustained and effective relief, coupled with its safety and patient-friendly design, underscores its promise as a valuable addition to the field of topical anti-inflammatory and analgesic therapies. Further research and clinical development are warranted to fully realize its potential in broader medical contexts.

## Figures and Tables

**Table 1:** Material composition of arnica extract hydrogel patch.

INCI Names	INCI Monograph ID	Composition (%)
Aluminum Glycinate	104	0.11
*Arnica montana* Extract	29,326	1.00
Cellulose Gum	457	2.30
Disodium EDTA	894	0.09
Glycerin	1077	39.00
Polyacrylic Acid	2402	2.00
Polysorbate 80	2457	0.10
Tartaric Acid	3146	0.30
Titanium Dioxide	3217	0.20
Water	3342	49.8
Sodium Polyacrylate	6285	4.80
1,2-Hexanediol	16,304	0.3

Arnica Patches were manufactured as shown above. pH conditions are 5.0 to 6.0. (Wooshinlabottach Co., Ltd., Seoul, Republic of Korea).

## Data Availability

There are no data associated with this study.

## Supplementary Materials

The following supporting information can be downloaded at: https://www.mdpi.com/article/10.3390/pharmaceutics17020171/s1, Table S1: q-RT PCR Primer Sequences for *il1b*, *il6* and *tnfa*.
