# Supplementary material for: Anti-Inflammatory and Pain-Relieving Effects of Arnica Extract Hydrogel Patch in Carrageenan-Induced Inflammation and Hot Plate Pain Models
Source: Pharmaceutics. 2025 Jan 28;17(2):171. doi: 10.3390/pharmaceutics17020171 (PMC11858859; doi:10.3390/pharmaceutics17020171)
Supplement: Supplementary file 1 [file pharmaceutics-17-00171-s001.zip › pharmaceutics-3364165-supplementary.pdf]

pharmaceutics

Supplementary Materials: Anti-Inflammatory and Pain-Relieving Effects of Arnica Extract Hydrogel Patch in Carrageenan-Induced Inflammation and Hot Plate Pain Models

Sang Gil Lee, Eun Byul Lee, Tack Soo Nam, Sunho You, Dahye Im, Kyusun Kim, Bonseung Gu, Ga-young Nam, Hyerim Lee, Soon Jae Kwon, Yun Seok Kim and Sang Geon Kim

Table S1. q-RT PCR Primer Sequences for *il1b*, *il6* and *tnfa*.

Gene.	Primer Direction	Sequence (5′ → 3′)
Mouse il1b	Forward	GGAGAACCAAGCAACGACAAAATA
	Reverse	TGGGGAACTCTGCAGACTCAAAC
Mouse il6	Forward	TTCCATCCAGTTGCCTTCTT
	Reverse	ATTTCCACGATTTCCCAGAG
Mouse tnfa	Forward	TCCCAGGTTCTCTTCAAGGGA
	Reverse	GGTGAGGAGCACGTAGTCGG
Mouse gapdh	Forward	AACGACCCCTTCATTGAC
	Reverse	TCCACGACATACTCAGCAC
